# The History, Value, Uses, and Effects of Quinine and Its Various Salts

**Published:** 1846-09

**Authors:** Samuel Henry Dickson

**Affiliations:** Professor of the Institutes and Practice of Medicine in the Medical College of the State of South Carolina; Charleston


### WESTERN JOURNAL

OF

### MEDICINE AND SURGERY.

---

SEPTEMBER, 1846.

---

### Art. I.—*The History, Value, Uses, and Effects of Quinine and its various Salts.* By Samuel Henry Dickson, M.D., Professor of the Institutes and Practice of Medicine in the Medical College of the State of South Carolina.

In compliance with the request of the East Tennessee Medical Society, I proceed to make a few remarks upon the History, Value, Uses, and Effects of Quinine or Quina and its various Salts. In the following pages I wish it to be understood, that my principal purpose is to collate and compare the views of others rather than to offer anything original on the subject. It would save any practitioner much time and enable him to avoid many errors, if he could obtain a clear statement of what his brethren had done and were doing, in regard to the management of the numerous forms of disease and the remedies employed for their relief and cure.

As in science and the arts generally, the earlier and more important discoveries were made in the East, so in medicine we derive from the same quarter of the globe some of our most impressive drugs. The use of opium alone, learned by the Crusaders from their Saracen enemies, may be regarded as a compensation, not inadequate, for all the evils inflicted upon Europe by this madness of the Middle Ages. The Asiatics, passing their lives in violent alternation between enthusiastic action and torpid repose, sought solace every where in the influence of powerful narcotics; and found in the gunjah and poppy the means of "steeping their senses in forgetfulness" and losing, in a blissful unconsciousness, the impatience natural under all sufferings.

When this western world was opened to the older sons of civilization, by the voyages of the two Italians, Colon and Vespucci, it soon hecame known that here also existed one narcotic worthy of being added to the resources of our restless race; and tobacco rose rapidly into favor in the Eastern hemisphere in spite of sage, moral, and royal denunciation. It is not however stated that, in the aboriginal practice of America, this weed has ever obtained a place; nor is it indeed a very great while since the bolder and more reasoning European has ventured upon its prescription in definite formulæ.

So far as I am aware, *Cinchona* is not mentioned at all by the first set of voyagers who landed upon the continent, nor have we any available information, either as to the period in which it came to the knowledge of the natives, or the mode in which they were led to its application to the cure of fevers. This last must be, from the nature of circumstances, a topic of unusual obscurity. In the narcotics of obvious influence above mentioned, accident, on the one hand, occurring during their habitual use, and on the other, a plain analogy, would point out their power to relieve pain and sooth restlessness, such frequent elements of disease. But cinchona, notwithstanding the absurdities affirmed by the homœopathists of the thousand and one symptoms to which it gives rise, is not in this way at all suggestive; and it would seem must owe to the merest chance its introduction into the Materia Medica. There is some doubt indeed whether this occurred to the aborigines at all. Vespucci, who gives an account of the manner of treating fevers resorted to by the Indians with whom he met, describes only their harsh methods of forcing an abundant sweat by rough exercise and vehement alternations of temperature. We are indebted to the Jesuit missionaries for the Peruvian bark, which their European experience may have led them to employ as a bitter tonic in febrile debility, and must not withhold from the good Fathers our thanks for this excellent boon to humanity. They have found their reward, doubtless, in Heaven; earth could offer nothing to recompense them for their saint-like beneficence and lofty self-devotion.

Cinchona soon grew to be a favorite with the profession on both sides of the Atlantic, and one is surprised at the readiness with which its application was extended from one form of disease to another, until it had been duly experimented with, in all or nearly all "the ills that flesh is heir to." This application became gradually more and more restricted, as a sort of gradual approach was made to the physiological notions (so called) that culminated and fell with Broussais; requiring us to refrain from stimulants in cases of hyperexcitement, and finding hyperexcitement almost everywhere. Universally regarded as a tonic and a stimulant, those who, with Brown and many of his predecessors and contemporaries, saw debility in all morbid action, were not apt to be deterred from its use even in fevers and inflammations.

Perhaps it was among West Indian practitioners, that there arose the two doctrines which gave a definite direction for a long while to the therapeutical employment of cinchona: I allude to the opinions even now prevailing, first, that it possesses an antidotal influence in contrast or opposition to that of the marsh poison, whatever it may be, malaria; and second, that it is essentially an antiperiodic, gifted with power to arrest all maladies of intermittent and paroxysmal recurrence. Consider how many pretexts became accumulated for the exhibition of bark. It was earliest introduced as a specific febrifuge, and prescribed in all fevers. It was next used by many as a tonic, and thus old Lieutaud recommends it in quotidians, because "besides its febrifuge virtue, *appertaining but little to this species of fever*, it obviously possesses a stomachic and roborant quality." Of course this adapted it to a long list of maladies. The most recent English writer on materia medica, Johnson, still asserts that "the great value of cinchona consists in the power which it possesses in arresting periodic or intermittent diseases." On this side the Atlantic, the profession, with great unanimity, hold to its antimalarial virtue. But McCulloch has taught us that masked, or obscure, or confused impressions from the malarial poison, may give rise to or modify almost all diseases of every class, order, and genus; and observation convinces us of the very general tendency to follow notable periods of remission and invasion or exacerbation.

Nor were our elder brethren slow in urging forward the remedy to obtain decided effects from large and impressive doses. Not to dwell on the practice of the West Indian physicians, the amount of half an ounce and an ounce was given even in London, by Huck and Fordyce. If the calculation be correct that five grains of quinine are equal to a drachm of bark in powder, we need not discuss, any longer, the question as to the merit of the first administration of large doses; *forty-five* grains of the former would satisfy most of our boldest practitioners.

But this dose was disgusting, offensive, and difficult to retain on the stomach, and we must congratulate ourselves and the world renewedly, upon the detection and separation of the alkaloid principle, in which resides the chief virtue of that Peruvian tree which has so long given health to the nations. I need not here say anything of the process by which Pelletier and Caventou were led to the discovery of this alkaloid and its convenient and invaluable salts. Honor to the men and country from whom we derive these gifts! Well do they deserve our gratitude and admiration. To France and to the French belong indeed the true glory of the highest civilization. Warlike in temper and of unlimited ambition, they yet feel, acknowledge and act upon the sentiment that the meed of fame does not appertain to courage only; but that the fairest laurels may be won as well by the skilful and devoted physician as by the brave soldier.—France and the French alone reward alike the arts of peace, and benevolence and preservation, with those of force, devastation and destruction.

The preparation of quinine in common use is properly a disulphate, though called universally the sulphate of quinine. It is nearly insoluble in water, but its solution is readily effected by adding a few drops of sulphuric acid, which converts it into the neutral sulphate and the bisulphate. There has been some question whether this quantity of acid may not exert an injurious influence upon the mucous surface of the digestive tube. This may easily be avoided I think; for I have not been able to perceive any difference of time between the effect of a solution of acid or neutral sulphate, and a diffused mixture containing the same quantity of disulphate in a little mucilage. The medicine, doubtless, passes rapidly into the circulating mass; for although it has not been detected in the blood, it is promptly found in the urine.

The specific nature of this important therapeutical agent; its essential tendency to affect certain organs or systems; to modify exclusively or peculiarly certain functions or sets of functions; these are topics of high interest, and have, especially of late, been widely and warmly discussed. I agree with those who rank it among the narcotics. Its action is eminently determined upon the brain and nervous system; and upon these it produces an impression altogether characteristic. A moderate dose given to a person in health, an overdose in the case of an invalid, will give rise to tinnitus aurium, a buzzing or rushing sound in the ears, combined with more or less deafness, a throbbing in the head, cephalalgia, occasionally some photophobia, and almost always, in greater or less degree, that state of mind and body which we call "nervousness," in which the hands tremble, the senses are readily startled by a sudden impression (as by an unexpected noise or jar), and the temper is petulant and irritable. The pulse in some persons becomes slower, in others it is more frequent.

The toxical or poisonous effects of quinine are very rarely met with among us. Having established the state or condition above described, now familiarly referred to in the use of the term quininism, few persist with the medicine or increase the amount exhibited. From authors we collect the following list:—vertigo; amaurosis; deafness; paralysis; prostration, as shown by smallness and rapidity of the pulse; coldness and paleness, or lividity of the surface; urticaria; cold and clammy sweats; cardialgia; nausea and vomiting; diarrhœa and dysentery; congestion of the lungs, as shown by dyspnœa and other signs; hematuria; a disposition to abortion in pregnant women; epileptic symptoms; delirium; coma; and in a few instances death. Some ascribe a portion of these accidents at least, as I have already said, to the amount of sulphuric acid requisite to the solution of the quinine. On the other hand, Briquet, in whose practice two of the fatal cases happened, lays great stress upon the completeness of this solution; dreading the accumulation of the insoluble disulphate in the digestive tube, and its sudden converson into a state of activity by some profuse secretion of acid. Briquet believes that when using the quinine in solution we shall always be safe, if we desist from if when prostration and vertigo appear, or the increasing frequency and smallness ot the pulse indicate a dangerous depression of the circulation. I have elsewhere entered into some details on this subject, and beg to refer to the Southern Journal of Medicine and Pharmacy, vol. i, No. i, for a history of two or three instructive cases.

It would be difficult to point out the fatal quantity of this agent. In one of the deaths occurring under the care of M. Briquet, the patient took "a comparatively small quantity of quinine;" in another not quite one hundred grains in two days; in a third, treated by M. Recamier, the fatal result took place from the exhibition of about one hundred and twenty grains, in doses of five grains every hour. Furious delirium with extreme agitation supervened, and death in a few hours; autopsy revealing an intense meningitis. We are accustomed in this country to the employment of *so much larger quantities* without injury, that we very readily admit the views and explanations of Briquet, who attributes the two first of the deaths spoken of, to other causes, developed on post-mortem examination, and considers the meningitis of the last to be an accidental coincidence. We shall see by and by that quinine has been used freely, and, as is alleged, successfully, in very serious inflammatory and quasi inflammatory affections of the brain and its membranes.

Of cinchona, either in powder, or infusion, or extract, there is no poisonous dose so far as we know. When an ounce or more of the powder has been taken, the stomach may be offended and reject it, but there are no worse consequences. Its bitterness is not very intense. Its tonic quality I am not inclined to doubt; but it is not clear that this belongs at all to the quina which it contains. Besides the cinchonine, whose influence is supposed to be closely analogous to that of quinine, it holds several vegetable principles together, of which, perhaps, the gallic acid and tannin may be the stimulant or excitant as well as astringent agents. In view of this varied composition of the drug, I am led to regret sincerely the disposition to abandon its use altogether; the indifference of the profession in procuring, selecting, and preserving it; and the almost universal desertion of nature's admirable combination of its constituents, for the elegant and invaluable preparation of one of its elements yielded us by art. I am persuaded that a proper discrimination should be attempted here; and that as there are still many severe maladies, in which we prefer to trust the crude opium rather than morphine and its delicate formulæ, so there are certain cases even of fever itself, which would be more decidedly benefited and would convalesce more thoroughly under some of the well chosen though almost obsolete modes of exhibiting cinchona, than the more recent and simple administration of quina and its salts.

I have already said that I regard quinine as a *narcotic.* This peculiar and vehement influence is clearly pointed out, both in its curative and poisonous effects. But this, it is contended is not all. Prof. T. Mitchell strenuously maintains its possession of a direct *febrifuge* or antifebrile power, and Professor Harrison agrees with him, that if anything can check or put an end to fever, quoad fever simply, it is this antidote. Its antiperiodic virtue is strongly insisted on by a large class of physicians, among whom we may select Cazenave as having adapted it to the treatment of diseases of almost indefinitely long intermission; and Miller who applies it equally to intermittent affections so short as to require nice observation. Of its *antimalarious* property I need say nothing; it is in our own country universally believed, and questioned by none any where except those who deny the separate existence of the marsh poison. Its *sedative* quality is by some supposed to be clearly made out—Boling and M'Cormick, with many others, have gone far to establish the fact, that it may be used with perfect safety, even where there are present the most decided symptoms of inflammation. Briquet has shown that it, at least occasionally, exerts an obvious power in controlling them. Its *anticongestive* agency, (I can contrive no better phrase) will find numerous advocates all over our south western country, where it is regarded as the principal or indeed almost the only remedy to be trusted to, in those attacks of fever specially characterized as congestive. If the enlargement of the spleen, whose relations to intermittent fever are by Piorry regarded as so essential, be of congestive form, they are asserted by him, whether transient or permanent, whether hypersplenohemia or hypersplenotrophia, to be under the most immediate and instantaneous subjection to quinine; a fact which may, if confirmed, be looked on as conclusive of this assumption.

I have thought that it may be of some advantage to collect together the several statements which in the course of my reading, I have met with, of its application to numerous and varying forms of disease. In the anxious search after remedies in difficult cases; in the doubtful hope mingled with eager wishes to preserve life and relieve suffering, which agitate always our most responsible profession, such a catalogue may be referred to as, at least, suggestive.

Of course it is proper to commence with *Intermittent Fever.* Its employment here is now universal, and the only mistake likely to be made by the merest tyro, is in the mode of administration of our very trustworthy remedy. In all ordinary cases, I prefer moderate, or what would perhaps be called, small doses, to large ones; a grain or two every second or third hour during the interval of our common tertians or quartans, will scarcely ever fail us; in the quotidian I would give four or five grains immediately on the subsidence of an exacerbation, and afterwards follow the same course as above laid down. In threatening attacks, pernicious, malignant congestive, the quantity should unhesitatingly be increased—the state of the nervous system already described as quininism, should be brought on as early as possible after the first paroxysm, and ought not to be permitted to subside entirely until two periods of invasion have passed by.

*Remittent Fever.* It used to be an acknowledged rule that we should wait for an entire intermission; but even the cautious practitioners of the old school made occasional exceptions—Lind, and Clark, and Irvine, employed cinchona in the best remissions they could obtain in persistent cases, after the first week or ten days. Fearne, of Huntsville, Ala., Tuck, of Memphis, Tenn., and Stratton, of Canada, may be quoted in favor of the beneficial administration of free and impressive doses of quinine in the management of remittent Fever.

*Continued Fever*, in all its forms, has been treated with cinchona and quinine; typhus and typhoid fevers were thus met by Huxham, Lieutaud, and many others of the old regime, who were forced to use the bark in substance, infusion or tincture. Recently, Waton and Briquet in France, and in our own country, Mitchell, Montgomery, and numerous fellow practitioners make use of the sulphate of quinine in all fevers known by these names.

*Yellow Fever.* The testimony on this subject is discrepant and deserves to be succinctly recounted. The ingenious and able editor of the New Orleans Journal, Prof. Harrison, speaks in strong terms of the success of Drs. Hunt, Mackie, and himself, in the yellow fever of 1839 in that city. The method to which he ascribes the favorable results "consisted in promptly giving the sulphate of quinine as soon as possible after the attack. The dose varied from 20 to 80 grains given in a little cold water. If the stomach was very irritable it was given by injection. If the first dose failed to produce an apyrexia in eight or ten hours, a second dose was given. The earlier it was given the better; but it should not be given after the second day." Thus late it always does injury. In this statement he differs from preceding authorities, by whom it is recommended either to wait for a natural apyrexia or remission, or to make one artificially by vehement V. S. or other modes of depletion. "I shall never forget my surprise," he says, "the first time I witnessed its effects. The fever in most cases was cut short as if by enchantment." From Dr. Watt, of Georgetown, Demerara, we learn that "the practitioners of that country give with great success in their yellow fever, 20 to 24 grains of sulphate of quinine with calomel every four hours until the symptoms abate." Beugnot, in an instructive essay in the first No. of the New Orleans Journal, has collected a host of authorities in that city and the West Indies, who give strong testimony in favor cinchona and quinine. To these we must add the names of Bally and Pariset, who saw sulphate of quinine highly serviceable in the yellow fever of Barcelona, in Spain, in 1820.

On the other hand, Dr. Lewis, of Mobile, reports an entire failure with it in all the various forms of yellow fever he met with, intermittent, remittent, and continued; and Dr. Stone speaks still more unfavorably of it. I have used several of the preparations of cinchona, with occasional benefit, in the yellow fever of Charleston, from twenty to thirty years since, as in the epidemics of 1817, 1819, and 1824; but its good effect was by no means constant, nor did the majority of cases admit of its employment at all. I was led to try it by the favorable reports of some of the West India physicians. In more recent epidemics I have experimented with the sulphate of quinine, but cautiously and in small doses. Not finding any good effect from it, I have abandoned it, though I still exhibit, in certain cases, the infusion of cinchona with the tincture, as "stomachic, roborant" and astringent.

*Continued Malarious Fever:* if there be such a disease, which I doubt, believing it to be an obscure remittent, complicated with serious congestions and inflammations, it is surely manageable with sulphate of quinine. Maillot describes such a fever in Bonao on the African coast, and Stratton at Penetarquishene on Lake Huron. Cordier gives us two cases of marsh fever (fievers marécagenses) occurring in the South of France, in which there was "complete impossibility of finding the slightest remission." They were promptly cured by sulphate of quinine.

*Congestive Fever.* I use this phrase in compliance with *custom* merely. In the condition which it implies, a condition common, doubtless, to all types of fever, American physicians almost universally regard sulphate of quinine as among the best remedies, if not the only one to be trusted to; and administer it in doses indefinitely large, from ℈i to ʒi or ʒii repeated pro re nata.

*Puerperal Fever.* Biagini, of Pistoria, recites the history of a case which his French translator, in the Journal des Connaissances, terms a "fievre perniscieuse, delirante, amaurotique," a malignant puerperal fever, complicated with delirium and blindness, "cured completely" by the use of the citrate of quinine.

*Hectic Fever.* I have seen the paroxysms of this fever of irritation, in a few instances, suspended by timely doses of sulphate of quinine, in moderate amount. In the great majority of the attempts I have made with it, however, it failed.

*Traumatic Fever.* Guersant reports its favorable influence in a patient for whom, in the Hôpital des Enfans at Paris, he had amputated both lower extremities.

*Erysipelas* is the only only one of the exanthemata admitting of introduction here. Its typhoid or contagious form as occurring in hospitals, has long been familiarly treated with free doses of bark, especially in London; and for my own part, I still prefer the infusion and tincture of cinchona to the quinine, which has superseded it among American physicians for the most part, in such examples as seem to allow of or indicate a resort to either.

Passing on from the pyrectic affections to the phlegmasiæ and others, *arachnitis* is affirmed to have been repeatedly treated by Bally with sulphate of quinine in the enormous dose of sixty-two grains.

*Encephalitis* is managed in the same way, though with smaller doses of our remedy by Delens.

*Epilepsy* was thus cured by Taroni in a young woman. The attack was caused by fright and manifested a marked periodicity. He began the treatment with 20 grains daily, gradually increased to 40 grains a day for six days, and then gradually diminished.

*Hemiplegia.* A very curious case of intermittent hemiplegia is told in the Journal des Connaissances for November 1st, 1845, which was treated skilfully and successfully by

Freschi, of Turin, with free doses of sulphate of quinine, after the failure of V. S., vesicatories, &c.

*Chorea.* Friberti reports a severe case, of two month's duration, caused by fright, in a child æt. 11 years, in which after a laxative, he gave a scruple of sulphate of quinine in twenty-four hours, in six potions. The course began on the 29th April; the child was well on the 12th May, and dismissed on the 15th.

*Hemicrania and Orbital* intermittent pains. Under this phrase Rodrique, of Montpellier, describes a very painful affection, often regarded as malarious, but, as I should judge, as often truly catarrhal, known by the names of 'brow-ague,' 'sun-headache,' &c. It is an intermittent inflammation of the mucous membrane of the frontal sinus and antrum. It is always regularly periodical; usually comes on in the forenoon and continues until near sunset. In Rodrique's case it came on every day at 3 o'clock, and lasted with severe suffering for four hours. He succeeded at last with free doses of sulphate of quinine, after failure with all other modes of treatment. I once met with similar success, but have seen the same treatment useless and inapplicable.

*Pneumonia.* In the pulmonary affections of malarious districts, cinchona in infusion, especially as combined with serpentaria and other diaphoretics, has been long considered a useful remedy. Drs. Boling, of Mobile, and Flint, of Buffalo, have presented strongly the claims of sulphate of quinine, as well adapted here; and McCormick says definitively that he has "seen it allay febrile action in pneumonia."

*Intermittent Dyspnœa* of great intensity, a paroxysmal strangulation (espice d'étranglement) which he oddly enough considers a "fiévre larvée," a masked or undeveloped fever, complicating hooping cough, is described by Morand. It attacked the patient, a child æt. 7 years, with many frightful symptoms of varying interest, at the same hour for four successive days, enduring each time a quarter of an hour. A dose of 60 centigrammes of sulphate of quinine put [illegible]

immediate end to the paroxysm, and another completed the cure.

*Rheumatism* has been already mentioned. Legroux administers the sulphate of quinine 'in small and divided doses.' Briquet in large amount. In acute rheumatism, the latter graduates the dose according to the intensity of the symptoms, "increasing the quantity in correspondence with the violence of the fever," exhibiting from 40 to 62 grains in the twenty-four hours. In chronic rheumatism, the doses were less; not exceeding 30 grains per diem, as a general rule. The first favorable effect, he says, is sleep; next, there is diminution of pain and swelling in the parts affected. He reports eight cases among twenty, cured in the short space of forty-eight hours.

Of *Splenic Enlargement*, hypersplenohæmia, or mere engorgement of vessels, and hypersplenotrophy, or ague-cake as it is termed with us, enough, perhaps, has been said.—Piorry, who maintains the universal dependence of intermittents upon splenic derangement, asserts the magical power of sulphate of quinine over all enlargements of this viscus; and even affirms that it is capable of producing within a few seconds, its total absorption when in a healthy state. These statments are incredible. We can scarcely doubt the influence of quinine and certain other agents to be hereafter mentioned, in causing the immediate subsidence of the spleen when the seat of simple sanguineous congestion; but is impossible that within 40 seconds, or even as many hours, absorption of solid tissue, whether the hypertrophy be analogous or heterologous, should take place, under the impression of any medicine whatever.

The unquestioned fact of the rapid disappearance of some of these splenic enlargements suggests the reasonableness of a trial of the sulphate of quinine in similar disorders of other vascular parts. *Orchitis*, for example, as we find it stated in the Journal des Connaissances, was treated by this remedy, successfully, after the failure of depletion, emollients, &c., in two cases under the care of Poura.

In the British and Foreign Review for June, 1845, there is a case of *constipation*, which, after obstinately lasting six days, was relieved by the sulphate of quinine in doses of five grains, repeated every half hour.

I conclude here this catalogue which might be almost indefinitely augmented from the periodicals on both sides of the Atlantic. If it convey a single hint, which shall aid an embarrassed practitioner in any difficult emergency, or contribute to the relief of a single fellow creature from danger or suffering, my purpose in its preparation will be fully attained.

We scarcely employ quina, in this country, in any other form than its combination with sulphuric acid; indeed its other salts are not easily procured. The *citrate* and the *lactate* are spoken of as milder and less offensive than the sulphate, while they retain all the requisite efficiency. The *valerianate*, which we owe to Prince Lucien Bonaparte, is highly lauded as combining very valuable antispasmodic and anti-irritative properties, with the rich virtues of the familiar article, but is scarcely accessible in ordinary practice, on account of its expensiveness. Devary assures us that it is equal to the sulphate in its antiperiodic effects, and superior in its nervo-sthenic properties. He reports many cases, some of which were severe and complicated intermittents, which the sulphate of quinine had failed to relieve, and which yielded when the valerianate was employed; he gave one grain at a dose and used per day from 1½ to 6 grains.—Meirieu recommends as preferable to the simple sulphate of of quinine a mixture containing that salt in solution and charged with an excess of carbonic acid gas; by which, he says, the quina is rendered more soluble, acts with greater energy and in a smaller dose.

Kingdon has prepared an *iodide* and *bin-iodide of quina*, with the view of "combining the tonic qualities of the one, with the excitement of the absorbents produced by the other of these powerful agents." He has exhibited the bin-iodide in several cases of scrofulous enlargement of the glands with great benefit.

The *ferro-cyanate* of quinine is very favorably mentioned by Dr. Fenner in the November, 1845, No. of the N. Orleans Journal, to which periodical I have made frequent reference as an abundant storehouse of useful facts and suggestions. He says, "it seems to exercise all the febrifuge and antiperiodic virtues" of the sulphate, without producing the disagreeable effects comprised under the phrase quininism, and that "it is much prescribed in cases where the head or stomach cannot bear the sulphate." An instructive case is detailed in which the ferro-cyanate was given in five grain doses every two or three hours, to a patient who refused to take the sulphate because it had made him perfectly deaf before. The condition of the subject was alarming; but the result was perfectly satisfactory; the head was not at all disordered and the paroxysm did not return.

Of all these combinations, however, that from which we would, a priori, expect most definite and certain advantage to be obtained, is the arsenite of quinine first found by Berzelius, as uniting two of our most impressive febrifuges. It is highly eulogized by Bertoloin, who confides in it almost exclusively, and has obtained with it successes equally astonishing by their rapidity and their permanence. He affirms that a single grain of this salt suffices to cut short the most obstinate relapses of intermittent fevers, and that its use not only cures the fever, but in the majority of cases dissipates all glandular enlargements. He thinks it prudent not to administer more than *one-twelfth* of a grain at a dose, although it has not proven injurious even in the amount of one-third of a grain.

Pereira and some others of the recent writers on Materia Medica warn us that the cinchona tree, being of native growth only over a very small territory, and capable of cultivation no where else, we are liable, in the process of even a short time, to have our supply of this most indispensable medicine limited most inconveniently, and even altogether exhausted. Our worthy Surgeon General, Lawson, has accordingly with laudable and sagacious forethought directed the attention of American practitioners to the search for substitutes, which may take the place of quinine, if we are in any manner deprived of it, and has particularly suggested the salicine as worthy of careful and extensive trial. The best report that we have as yet on the subject, is that of Dr. Fenner, one of the editors of the New Orleans Journal, who gives the result of 20 cases of fever treated with salicine. Its great inferiority to quinine will not be denied by any one, but both from the very valuable details afforded us by Dr. F., and from a few observations I have myself had an opportunity of making, I am entirely satisfied that it possesses decided febrifuge properties.

*Opium* and many of the preparations of its constituent elements, ought to be considered as among our best aids to quinine, and also among our most promising substitutes for it. The *muriate of narcotine* eulogized by O'Shaughnessy is unequivocally an antiperiodic of very considerable power.

Dr. Corrigan gives us a case of ague in which sulphate of quinine had been administered "with the effect only of prolonging the intervals between the fits. A dose of *sulphuric ether*, ℨii, with tinct. opii $g^{tt}$.xx, put an end to the attack. The spleen in five minutes diminished from 6 inches by 7½ inches to 4½ by 6¾; the skin becoming warm (it was in the cold stage) and the pulse fell from 120 to 96." Hence Dr. C. concludes—"1. That the spleen can suddenly alter its volume. 2. That other agents as well quinine can produce this sudden alteration and size; and 3. That the cure of the disease can be effected by other remedies." The meaning of this last proposition is evidently that sulphuric ether and opium are available substitutes for quinine, an assertion which we would gladly see confirmed by experience.

*Piperine* must not be omitted in this enumeration of our armory of febrifuges, in case of an unfortunate failure of the supply of quinine and cinchonine or cinchona. It is an adjuvant of great value, not only when we are employing these latter, but combines well with any on the list of substitutes for it.

I should not have thought it necessary to refer in this connection to *arsenic*, so well known and familiar an alterative, preferred by some and by most considered only second to quinine, but that I wished to draw attention to some remarks published not long since, by M. Boudin, Med. en chef de l'Hôpital Militaire de Versailles. This gentleman whose experience seems very extensive, tells us that he has substituted arsenic altogether for quine, as far more certain in its effect and more worthy therefore of our confidence. He treats all his cases with it, and has met with uniform success; he affirms that the sulphate of quinine had previously been given to more than five hundred of his patients in vain.

In some of his views, I am disposed to agree with him, while others I look upon as erroneous. I prefer to commence the management of every case with the quinine, but, like him, I have thought the impressions made by it, however efficient and salutary, less permanent than those which arsenic produces on the system. I have had frequent occasion to witness the return of intermittents cured by quinine alone, after longer or shorter intervals; and this recurrence becomes very annoying to some patients. But I have never yet seen a relapse or return of the disease, unless after new and obvious exposure to its miasmatic sources, after the arsenical treatment had been fairly carried out.

Lastly, I must say a few words of *bebeerine*, the most promising by far of all the substitutes proposed for our invaluable remedy; and indeed if the reports already received, prove correct and are established by a prolonged and extended experience, one destined to become a formidable rival to it whenever we are permitted a choice. Its claims are well supported by Dr. M. Logan in the Edinburgh Medical and Surgical Journal. It is a vegetable alkali, obtained from a plant of the natural order *Lauraceæ*, genus *Nectandra*, called

*N. Rodiei*, in honor of Dr. Rodie, the discoverer of its medicinal powers. Like quinine it is prescribed in its combination with sulphuric acid, as a disulphate or a sulphate. It is sold at less than half the price of the disulphate of quinine, its dose being about 12 grains, in which quantity it does not disorder the head or produce any other unpleasant effects. One authority tells us that it acts occasionally as a laxative. Dr. Watt, of Demarara, thinks it, like arsenic, slower but more permanent in its influences than quinine, from whose accidental or toxical annoyances it is quite free. Dr. W. is subject to urticaria when he takes quinine and resorts to bebeerine preferably, as not exciting any such annoyance. In a case of symptomatic fever from abscess, the patient took with good effect sulphate of quinine in 10 grain doses, but he also was distressed with a consequent urticaria; substituting bebeerine in the same dose, he obtained the same benefit without the nettle rash. Dr. J. Hughes Bennett, of Edinburgh, a very respectable authority, declares that he has "no doubt that bebeerine possesses all the valuable properties of quinine in an equal if not a superior degree," Prof. Simpson, aware that Piorry accuses quinine of being apt to produce abortion or premature labor, tried the sulphate of bebeerine in the periodic neuralgia of pregnant women, with perfect success; he regards it as "antiperiodic and tonic, with far less stimulant and irritating effect, than we see produced by quinine.

As yet, I have been unable to procure any of this highly lauded medicine, nor do I know that any of it has hitherto been imported into our country. As soon as it can be obtained, I shall eagerly resort to it, and if not disappointed in my hopes of benefit from it, shall gladly add it to the brief list of remedies regarded with trust and confidence.

Charleston, April 11th, 1846.

